# Respiratory Syncytial Virus Infections in Polish Pediatric Patients from an Expert Perspective

**DOI:** 10.3390/vaccines11091482

**Published:** 2023-09-13

**Authors:** Maria K. Borszewska-Kornacka, Agnieszka Mastalerz-Migas, Aneta Nitsch-Osuch, Teresa Jackowska, Iwona Paradowska-Stankiewicz, Ernest Kuchar, Jan Mazela, Ewa Helwich, Marcin Czech, Ryszard Lauterbach, Jarosław Pinkas, Mirosław Wielgoś, Jacek Wysocki

**Affiliations:** 1 Coalition for Preemies Foundation, Medical University of Warsaw, 02-091 Warsaw, Poland; 2 Department of Family Medicine, Wroclaw Medical University, 50-368 Wroclaw, Poland; agnieszka.migas@gmail.com; 3 Department of Social Medicine and Public Health, Medical University of Warsaw, 02-007 Warsaw, Poland; anitsch@wum.edu.pl; 4 Department of Pediatrics, Centre of Postgraduate Medical Education, 01-813 Warsaw, Poland; tjackowska@gmail.com; 5 Department of Epidemiology, Infectious Diseases and Surveillance, National Institute of Public Health—National Institute of Hygiene—National Research Institute, 00-791 Warsaw, Poland; istankiewicz@pzh.gov.pl; 6 Department of Pediatrics with Clinical Assessment Unit, Medical University of Warsaw, 02-091 Warsaw, Poland; ernest.kuchar@gmail.com; 7 Department of Neonatology, Poznan University of Medical Sciences, 60-535 Poznań, Poland; janco@pol-med.com.pl; 8 Institute of Mother and Child, 01-211 Warsaw, Poland; sekretariat.neonatologii@imid.med.pl; 9 Polish Pharmacoeconomic Society, Institute of Mother and Child, 01-211 Warsaw, Poland; marcin.czech@imid.med.pl; 10 Polish Neonatal Society, Clinical Department, University Hospital in Krakow, 30-688 Kraków, Poland; ryszard@lauterbach.pl; 11 Centre of Postgraduate Medical Education, 01-813 Warsaw, Poland; jjpinkas@gmail.com; 12 Medical Faculty, Lazarski University, 02-662 Warsaw, Poland; miroslaw.wielgos@gmail.com; 13 Department of Health Prevention, Faculty of Health Sciences, Poznan University of Medical Sciences, 61-701 Poznań, Poland; jwysocki@ump.edu.pl

**Keywords:** disease burden, epidemiology, respiratory syncytial virus, seasonality

## Abstract

Respiratory syncytial virus (RSV) is the most common pathogen causing respiratory tract infections in infants, affecting over 90% of children within the first two years of life. It may cause lower respiratory tract infections, which constitute a significant healthcare burden both in the primary and secondary care settings. Meanwhile, the data regarding RSV disease in Poland is scarce, and published data significantly differs from the numbers reported for other countries with longstanding surveillance and reporting systems. A literature review and an expert panel were conducted to (1) understand the healthcare burden of RSV infections in Poland; (2) collect data on infection seasonality, patient pathway, and management patterns; and (3) evaluate RSV infection surveillance in Poland. According to the literature, RSV is the major agent responsible for non-influenza respiratory diseases in Poland. The reported rates of hospitalization for RSV infections are 267.5/100,000 for children under 5 years of age and 1132.1/100,000 for those under 1 year of age. Comparisons with data from other countries suggest that these values may be underestimated, possibly due to insufficient access to microbiological testing and a low awareness of RSV. Infections occur mainly between December and April, however, this pattern has changed following the implementation of preventive measures for coronavirus disease 2019 in the past few years. According to available reports, bronchodilators, antibiotics, corticosteroids, and X-ray imaging have been frequently used. The surveillance system in Poland has limitations, but these may be overcome due to recent changes in healthcare law as well as the availability and reimbursement of diagnostic tests.

## 1. What Is Known

The most common pathogen responsible for acute respiratory tract infections in children is respiratory syncytial virus (RSV), and it is the leading cause of infant hospitalizations and the second leading cause of infant death after malaria. 

The management of RSV infections remains a significant challenge for the healthcare system.

## 2. What Is New

This position paper summarizes the most important information on RSV disease in Poland with the aim to help assess the appropriate level of resources and develop preventive strategies.

## 3. Introduction

Acute respiratory tract infections (ARTIs) are among the most frequent causes of hospitalizations and visits to follow-up departments in children. One of the most common pathogens responsible for ARTIs in children is respiratory syncytial virus (RSV) [1,2]. The exact frequency of RSV infections is unknown because some cases are asymptomatic [3]. However, it is estimated that over 90% of children are infected with RSV by the age of 2 years [4,5] and that the virus is responsible for approximately 20% of ARTI cases [6]. In 2019, the global number of RSV-associated acute lower respiratory infection episodes was estimated at 33 million and the number of RSV-associated acute lower respiratory infection hospital admissions—at 3.6 million [7]. The clinical presentation of RSV infection can vary widely, ranging from asymptomatic disease and upper respiratory tract symptoms to lower respiratory tract disease, where patients may develop bronchiolitis, pneumonia, or bronchitis [4,8]. The risk factors for severe RSV infection include prematurity, low birth weight, chronic lung disease, congenital heart disease, artificial nutrition, attendance at daycare centers, crowded living conditions, presence of school-age siblings at home, and exposure to secondhand/passive smoke [8,9]. However, most children hospitalized due to RSV infection have no history of prematurity or underlying medical conditions [10,11,12].

The management of RSV infections remains a significant challenge for the healthcare system. Therefore, it is essential that stakeholders responsible for implementing systemic solutions have a good understanding of the exact disease burden as well as aspects of disease management, prevention, and surveillance. Meanwhile, the data regarding these aspects of RSV disease in Poland is scarce. Most of the existing publications are single-center studies. Published data significantly differ from numbers reported for other countries with longstanding surveillance and reporting systems. Thus, the current study was designed to summarize the most important information on RSV disease in the pediatric population in Poland with the aim to help assess the appropriate level of resources and develop preventive strategies. Specifically, our goals were: (1) to understand the healthcare burden of RSV infections in Poland; (2) to collect data on infection seasonality, patient pathway, and management patterns; and (3) to evaluate RSV infection surveillance in Poland.

## 4. Methodology

To map all accessible information regarding RSV in Poland and fulfill the goals mentioned above, a literature search was conducted in April 2023 in PubMed and Cochrane databases, using the following search terms: (“Respiratory Syncytial Virus” OR RSV OR bronchiolitis) AND (Poland OR Polish) AND (“disease burden” OR “clinical burden” OR “economic burden” OR epidemiology OR surveillance OR mortality OR morbidity OR incidence OR infection OR consultation OR “hospital admission” OR hospitalization OR “intensive care unit” OR death OR cases OR “attack rate” OR “direct costs” OR “indirect costs” OR absenteeism OR “psychological impact” OR “hospital saturation” OR “antimicrobial resistance” OR ARI OR SARI OR ILI OR “risk factor” OR sequelae OR asthma OR wheezing OR allergies OR seasonality). Only papers published between 2010 and the first quarter of 2023 in English or Polish language, describing the pediatric population were included, while case studies were excluded. The search yielded 78 records, of which 26 were considered to report meaningful data for Poland. The chosen papers were then classified based on topics they provided information on and used in the appropriate sections of the review.

Next, an expert panel, consisting of the authors of this article, was convened. The expert panel included the main Polish specialists in infectious diseases, pediatrics, neonatology, perinatology, vaccinology, epidemiology, and public health. The experts were scientists with international achievements and national consultants or heads of scientific societies in the above fields with an important impact on the health system in Poland. They prepared parts of the publication according to their competencies and data they could share due to their functions.

## 5. Results 

### 5.1. The Burden of RSV Disease in Poland

#### 5.1.1. RSV Symptoms and Diagnosis 

An RSV infection can be confirmed by antigen or polymerase chain reaction (PCR) testing. Unlike PCR, antigen tests can only detect high viral load, so asymptomatic cases and those with a low amount of the virus can be better detected using PCR. However, in many cases, the exact etiology of ARTI remains unknown. The most common clinical forms of RSV infection are bronchiolitis, bronchitis, and pneumonia. Symptoms include rhinorrhea, cough, sneezing, low-grade fever, and wheezing in mild disease, while in more severe cases, patients present with an increased respiratory rate, intercostal and subcostal retractions, hyperexpansion of the chest, restlessness, and peripheral cyanosis. In the most severe form, patients develop central cyanosis, tachypnea of more than 70 breaths/min, lethargy, and apneic spells, and the disease is life-threatening [9]. There is currently no targeted therapy, and the treatment remains supportive.

#### 5.1.2. RSV-Related Hospitalizations

The burden of respiratory diseases in Poland was estimated in a study by Lange et al., assessing all hospitalizations due to ARTIs in 2014 [13]. In the whole pediatric population, ARTIs were responsible for 32.4% of all hospitalizations. In a total number of 101,000 children hospitalized for ARTI, bronchitis, and bronchiolitis (International Classification of Diseases, Tenth Revision [ICD-10] code range J20-J22—Other acute respiratory infections), ARTIs constituted 30% of cases (30,500 patients). Children and adolescents accounted for 70% of all hospitalizations due to bronchitis and bronchiolitis. Bronchiolitis (ICD-10 code J21) was diagnosed in 1754 children younger than 1 year of age, 196 children aged 2 to 5 years, and 11 children older than 6 years. The incidence rates for bronchitis were 2633.4 per 100,000 for patients younger than 1 year, 1220.3 per 100,000 for children aged 2 to 5 years, and 221.9 per 100,000 for children older than 6 years (for adults, this rate was 108.9 per 100,000). The incidence of bronchiolitis in the youngest age group was 238.3 per 100,000 [13].

The proportion of RSV-positive results in pediatric patients hospitalized for ARTI ranged from 31% in children younger than 2 years to 49–53% in those younger than 5 years [14,15,16]. Pancer et al. [16] concluded that RSV was the main cause of ARTIs (mostly lower respiratory infections) and hospitalizations in children. Most patients with RSV infection hospitalized due to ARTI were children younger than 1 year—they constituted 94–96% of all RSV-positive cases [14,17]. Of all positive samples, 65–73% were obtained from patients younger than 6 months [14,15].

In a population-based study, Rząd et al. [4] assessed 57,552 hospitalizations for RSV infection between 2010 and 2020 among children younger than 5 years. The estimated hospitalization rate in this population was 267.5 per 100,000 (1132.1 per 100,000 for children younger than 1 year). Most patients were children aged less than 1 year (81.7% of cases), of whom 61.7% were infants up to 6 months of age [4]. Hospitalization rates in this study were lower than those reported by other authors. For example, in Australia, the rate was 490 per 100,000 among children younger than 5 years, while an analysis of data from 32 countries for the same age group showed a rate of 437 per 100,000 [18,19]. For European Union countries estimated hospitalization rate of children up to 5 years was 1006 per 100,000 [20]. According to the literature, the mean duration of hospitalization for RSV infection ranges from 5.5 to 11.2 days [14,21,22].

**Figure 1:**
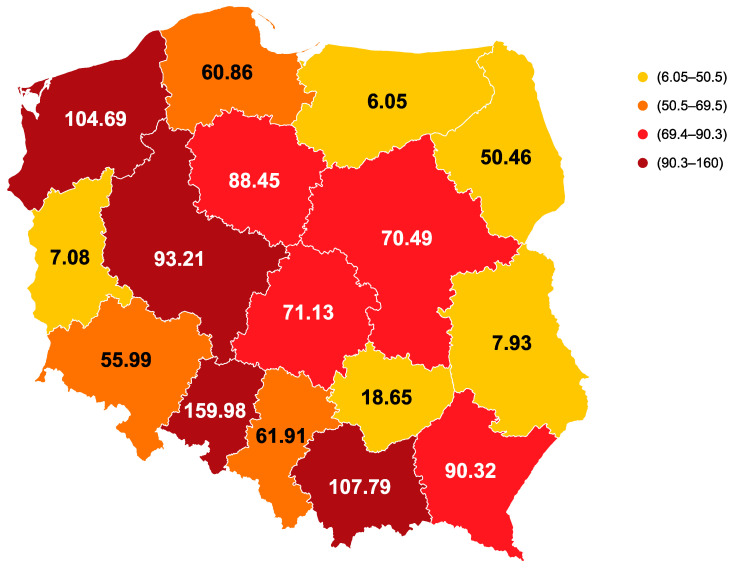
Number of hospitalizations due to RSV infections per 100,000 children [23].

The number of hospitalizations due to RSV infections (ICD-10 codes J12.1, J20.5, and J21) in relation to the size of the pediatric population differed considerably between voivodeships in Poland, with values ranging from 6.05 to 159.98 per 100,000 (Figure 1) [23].

According to epidemiological data collected before the coronavirus disease 2019 (COVID-19) pandemic in some other countries, RSV infections were most common among the youngest pediatric patients. However, more recent data reveal a slight shift in this trend, with older children being more commonly affected by the disease [24,25,26,27]. Data showing similar trends for Poland are currently lacking. 

#### 5.1.3. Outpatient Burden of RSV Disease

In contrast to hospitalizations, data on RSV infections in the outpatient setting are scarce. The reason for this is financial: hospital procedures are priced higher in Poland if the etiology is known. On the other hand, until recently, testing for RSV infection in outpatient health centers has not been financed from public funds, so there has been no incentive to provide such services. Therefore, RSV tests were not common in the outpatient setting, even though viral testing of children with bronchiolitis was recommended for epidemiological reasons and to lower the unnecessary use of antibiotics [21]. As a result, the etiology of up to 50% of ARTI cases per year remained unknown [16].

The limited data on RSV infections suggest that among non-influenza viruses detected in positive samples (constituting approx. 50% of all positive samples), RSV is the predominant one, accounting for over 96% of cases among children aged 0 to 14 years [28,29] and 55% to 91% of cases among the general population (Table 1 [30,31,32,33,34,35]). Therefore, RSV is the major agent responsible for non-influenza viral respiratory infections in Poland [31,36].

Between 1 January and 30 April 2023, a total of 1361 RSV infections and 700 hospitalizations for RSV disease were reported, including 676 infections and 517 hospitalizations among children younger than 2 years of age [37]. 

#### 5.1.4. Patient Pathway

**Figure 2:**
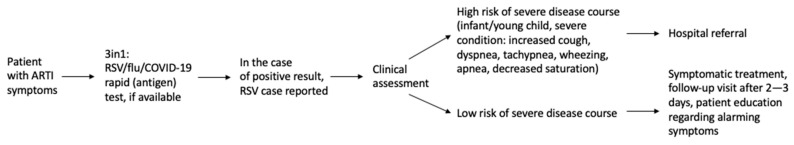
Patient pathway in the outpatient setting. Abbreviations: ARTI—acute respiratory tract infections; COVID-19—coronavirus disease 2019; RSV—respiratory syncytial virus.

**Figure 3:**
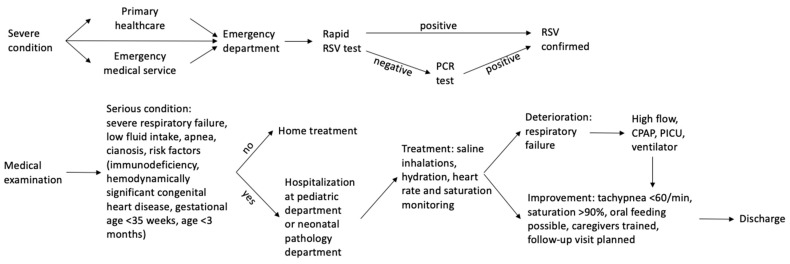
Patient pathway in the hospital setting. Abbreviations: CPAP—continuous positive airway pressure; PCR—polymerase chain reaction; PICU—pediatric intensive care unit; RSV—respiratory syncytial virus.

In recent years, partly due to the COVID-19 pandemic, new diagnostic tools have become available for general practitioners and pediatricians who deal with ARTIs. While rapid PCR and strep tests allow clinicians to differentiate between viral and bacterial infection and guide therapeutic decisions on antibiotic prescription, the combo test for COVID-19, influenza, and RSV distinguishes different viral pathogens. Its results affect the reporting of cases, while a decision regarding potential hospitalization is based mostly on clinical assessment of risk severity (Figure 2 and Figure 3).

### 5.2. Management of RSV Infections

Currently, there is no effective treatment for RSV-related infections, and the management is only supportive [38]. According to recommendations, antibiotics, bronchodilators, and corticosteroids should not be routinely applied. Corticosteroids can be considered in patients with significant airway obstruction, and β_2_-agonists may be used in individual cases. Inhaled hypertonic saline can be applied in selected hospitalized patients. Oxygen therapy is recommended if the oxygen saturation is lower than 90% [39], although recent studies clearly show that high-flow nasal cannula (HFNC) therapy is more effective than passive oxygen therapy [40,41,42]. The results of chest X-ray imaging do not correlate with disease severity. The use of chest radiography leads to increased use of antibiotics, so it should be limited to patients suspected of serious respiratory complications or considered for intensive care unit admission [39,43].

Several studies showed low compliance with the recommendations (Table 2) [14,22,44,45,46,47,48,49]. The use of bronchodilators was reported in more than 85% of patients in all studies; antibiotics were prescribed in 16% to 100% of patients, inhaled corticosteroids in 12.2% to 87.5%, and systemic corticosteroids in 6.1% to 55.7% of patients. Chest X-ray imaging was used in 31.6% to 95.3% of patients [14,22,44,45,46,47,48,49]. Antibiotics are usually used to prevent bacterial superinfections, even though they are sporadic in RSV cases [14]. A trend toward decreasing the use of antibiotics and X-ray imaging was reported [47,48].

### 5.3. Prevention of RSV Infections

Currently, there is no effective vaccination against RSV infections in children. The only available product that prevents lower respiratory tract disease caused by RSV in this population is a humanized mouse monoclonal immunoglobulin G1, palivizumab (Synagis). It is administered in a series of 5 intramuscular injections every 30 days during the RSV season at a dose of 15 mg/kg of body weight [50]. In Poland, palivizumab has been used since 2008 in children with a high risk of severe disease (Table 3) under a drug program available in 67 tertiary neonatal centers. 

According to the expert’s statement, palivizumab immunoprophylaxis should also be provided to infants younger than 2 years of age who have cystic fibrosis and neuromuscular disorders [51]. 

Emerging therapies may change the prevention of RSV infection. In November 2022, the European Commission approved Beyfortus (nirsevimab), a human immunoglobulin G1 kappa monoclonal antibody, for the prevention of RSV lower respiratory tract disease in newborns and infants during their first RSV season [52]. In a study including 1490 infants, medically attended RSV-caused lower respiratory tract disease occurred in 1.2% of children in the nirsevimab group compared to 5.0% in the placebo group, translating into an efficacy of 74.5%. These results proved that a single dose of nirsevimab effectively protects healthy late preterm and term infants from medically attended RSV-associated lower respiratory tract infections [53]. Nirsevimab was recommended to all children younger than 6 months by Spanish and French experts [54,55]. A recent systematic review and meta-analysis demonstrated that both palivizumab and nirsevimab significantly reduce the risk of RSV disease and severe disease course compared with placebo [56]. 

In April 2023, the European Medicines Agency (EMA) recommended a marketing authorization in the European Union for Arexvy, the first vaccine to protect adults aged 60 years or older against lower respiratory tract disease caused by RSV. In July 2023, Pfizer’s Abrysvo (a bivalent subunit vaccine) was approved by EMA for use in older adults and pregnant women. One more vaccine candidate against RSV is currently in late-phase clinical trials: an mRNA-based vaccine by Moderna. Each of these vaccines is applied as a single-dose intramuscular injection and has been shown to have a good safety profile [57]. 

### 5.4. Epidemiology and Surveillance of RSV in Poland 

#### 5.4.1. Seasonality of RSV Disease

The majority of RSV infections in Poland are identified between January and March, with December and April following closely behind [4,14,15,16,17]. Only 8.4% of cases were reported in other months [15]. The analysis of data from 8 seasons suggested that the epidemic season most often starts at week 50 (late December) and lasts until week 15 of the subsequent year (half of April), reaching its peak between weeks 4 and 10 (February and March) [17]. 

The emergence of COVID-19 prompted the implementation of preventive measures to limit the spread of severe acute respiratory syndrome coronavirus 2. This led to variations in the circulation of common respiratory viruses, including RSV. After the initial decline of cases, when restrictions were lifted, a shift in seasonality and delayed outbreaks of RSV were observed in several countries [24,25,27,57,58,59,60,61,62]. This pattern was confirmed by Polish investigators, although data are limited. In one study, a similar number of hospitalizations due to RSV respiratory tract infections was recorded in the seasons 2018–2019 (n = 35), 2019–2020 (n = 56), and 2021–2022 (n = 43), while in the 2020–2021 season, there were no RSV-positive patients [44]. In another single-center study, no hospitalizations due to RSV were recorded between October 2020 and June 2021. However, the first cases were noted in July 2021, and the number of hospitalizations increased over the subsequent months, reaching a peak in October when 69% of hospitalizations took place [22]. 

**Figure 4:**
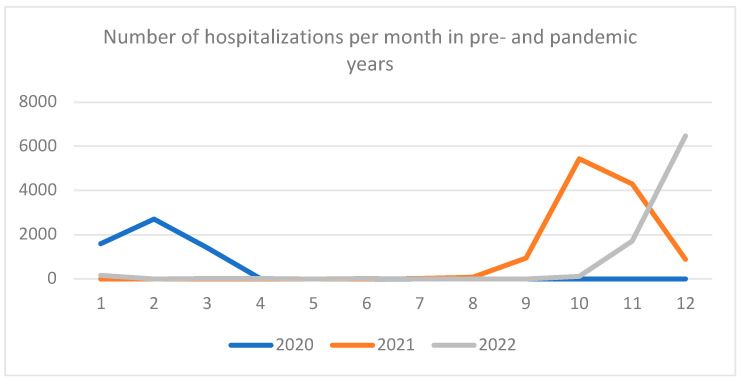
Number of hospitalizations due to respiratory syncytial virus infections between January 2020 and December 2022.

Data from the National Health Fund regarding hospitalization rates in children younger than 2 years of age hospitalized due to RSV-related ARTI (ICD-10 codes J12.1, J20.5, J21.0, and B97.4) in the seasons 2020–2021 and 2021–2022 show a change in the seasonality pattern in 2021 and a significant increase in the number of hospitalizations in both seasons (Figure 4).

**Figure 5:**
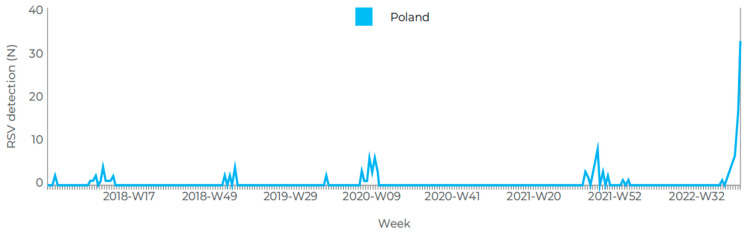
The number of cases of respiratory syncytial virus (RSV) infections in Poland since the 2017–2018 season (generated from: ECDC/Surveillance Atlas of Infectious Diseases, reprinted from Konkret24 [63]).

The same pattern can be seen in the data reported to the European Centre for Disease Prevention and Control for the seasons from 2017–2018 to 2022–2023 (Figure 5). The weekly number of reported cases in mid-December 2022 was 36, as compared with 4 to 8 cases per week in the previous years [63].

#### 5.4.2. Surveillance of RSV Infections

In Poland, all cases of ARTIs are reported every week both by outpatient care centers and by hospitals. Moreover, since February 2023, outpatient departments have been obliged to record RSV infections confirmed by laboratory testing. Until recently, this was required only for influenza and COVID-19. The change was prompted by the increased availability of rapid RSV tests and the fact that their use has been covered by the National Health Fund since January 2023. Information on the incidence of RSV infections is provided also by the influenza surveillance system called Sentinel, whereby a network of general practitioners and regional sanitary and epidemiological stations test for influenza, COVID-19, and RSV infections. The number of reported cases is published once a week on the website of the National Institute of Public Health. 

Before implementing the above advances, there was no widespread testing for RSV or obligation to report RSV cases. Therefore, past data are incomplete and underestimated. As RSV was included on a list of alert pathogens, it had to be entered into the registry of alert pathogens if detected in the hospital. However, only local analyses were available, and there were no official country-wide data on the spread of infections. In addition, the specific burden of RSV (i.e., the number of positive RSV cases out of all ARTI cases) was not reported. Moreover, as available reports did not specify whether medical services were related to RSV or other respiratory viruses, there was no information on the severity of RSV disease. Finally, pediatricians may have been underrepresented in the surveillance network, resulting in an underestimation of RSV cases among pediatric patients. However, recent changes in healthcare law and the availability of rapid testing provide an opportunity to improve RSV surveillance. 

## 6. Discussion

This is the first publication widely describing several aspects of RSV disease in children below 2 years of age in Poland. RSV is the main cause of ARTIs and pediatric hospitalizations in Poland, particularly among children below 1 year of age. Managing RSV infections is a considerable challenge to the healthcare system. As such, it is crucial to find comprehensive solutions and possess a thorough comprehension of the precise disease burden, along with various facets of disease management, prevention, and surveillance. However, the available data concerning these facets of RSV disease in Poland remains limited. It seems that the rates for Poland were underestimated, which can be explained by insufficient access to microbiological testing and low awareness of RSV [4,64]. There are significant disparities between voivodeships in Poland concerning the number of hospitalizations attributed to RSV infections [23]. These variations suggest differing diagnostic capabilities among regions, as well as the absence of a standardized surveillance system. 

As RSV stands as the primary causative agent for non-influenza viral respiratory infections in Poland [31,36], tests in an outpatient setting were not consistently conducted. As of January 2023, the National Health Fund in Poland covers the cost of rapid antigen testing for influenza, COVID-19, and RSV in outpatient departments. Testing is recommended for patients with respiratory tract infections who are at risk of severe disease course, including children younger than 5 years, people older than 65 years, pregnant women, and patients with chronic diseases, obesity, and immunosuppression.

Particularly noteworthy is the low compliance with the recommendations on the management of RSV infection. It may be the result of the absence of Ministry of Health regulations and official recommendations from scientific societies as well as the lack of targeted treatment, only symptomatic treatment is currently used in patients with RSV infections. Therefore, treatment usually differs depending on the physician’s experience and it often leads to excessive usage of antibiotics, corticosteroids, and X-ray imaging. 

There is clear seasonality of RSV infections with epidemic season lasting from December to April [56,65] with its peak between February and March which is in line with European data [66,67]. Understanding the seasonality of RSV infections is important for planning prevention strategies and predicting healthcare resources necessary to deal with an increased number of cases during disease outbreaks. Therefore, further studies are needed to investigate RSV seasonality after the COVID-19 pandemic. The recent expansion of the palivizumab drug program and the emergence of new prevention options create a chance for the effective protection of a bigger population. New regulations regarding RSV testing, along with updated financial rules, made easier access to RSV testing. Additionally, the implementation of new reporting rules is expected to enhance RSV surveillance in Poland. Furthermore, there remains a need to develop optimized and uniform recommendations for the clinical management of RSV infections, both in primary and specialist care facilities.

## Figures and Tables

**Table 1:** Proportions of RSV-positive samples in all positive non-influenza samples in an outpatient setting.

Publication	Epidemic Season	Proportion of RSV-Positive Samples in All Positive Non-Influenza Samples	Population
Kondratiuk et al. [31]	2013/14	84%	General
Hallmann et al. [29]	2014/15	96.2%	Pediatric
Woźniak-Kosek et al. [30]	2010	66.7%	General
Czarkowski et al. [35]	2011/12	87.5%	General
Bednarska et al. [33]	2012/13	87%	General
Byambasuren et al. [34]	2008/09	54.6%	General
Byambasuren et al. [34]	2009/10	56.7%	General
Byambasuren et al. [34]	2010/11	74%	General
Byambasuren et al. [34]	2011/12	86.7%	General
Byambasuren et al. [34]	2012/13	87%	General
Byambasuren et al. [34]	2013/14	84.5%	General
Byambasuren et al. [34]	2014/15	91.4%	General
Byambasuren et al. [34]	2015/16	77.5%	General
Byambasuren et al. [34]	2016/17	87.5%	General
Byambasuren et al. [34]	2017/18	63.1%	General

**Table 2:** Reported rates of using selected therapies in patients with respiratory syncytial virus infections.

Study	Inhaled Corticosteroids	Systemic Corticosteroids	Bronchodilator	Antibiotics	Chest X-ray	Supportive Care Only
Pogonowska et al. [22]	85.2%	55.7%	90.2%	100%	NA	NA
Fedorczak et al. [44]	60.5%	25.6%	88.4%	44.2%	NA	2.3%
Cegielska et al. [14]	66%	34%	93%	69%	75%	NA
Sosnowska et al. [45]	12.2%	6.1%	NA	31.6%	31.6%	NA
Haładaj et al. [46]	87.5% (budesonide)	NA	NA	93.7%	NA	NA
Wrotek et al. [47]	NA	NA	NA	16%	NA	NA
Wrotek et al. [48]	NA	NA	NA	NA	61%	NA
Kusak et al. [49]	62.1%		86.4%	62.1%	95.3%	NA

Data are presented as percentages of patients. NA, not applicable.

**Table 3:** Changes in the palivizumab drug program since 2008.

Seasons	Inclusion Criteria
2008–2009	Children with BPD:
	Born in 2008, gestational age ≤ 30 weeks, or Born in 2007, gestational age ≤ 26 weeks, or Aged < 2 years with severe BPD
2009–2013	Children with BPD:
	Aged < 3 months, gestational age < 30 weeks, or Aged < 6 months, gestational age < 28 weeks
2013–2018	Children aged < 1 year and:
	Gestational age ≤ 28 weeks, or BPD
2018–February 2023	Children:
	Aged < 1 year and: - gestational age ≤ 28 weeks, 6/7 days, or - BPD Aged < 6 months and gestational age 29–32 weeks, 6/7 days
Since March 2023	Children:
	Aged < 6 months and: - gestational age 29–32 weeks, 6/7 days, or - gestational age ≤ 35 weeks, 6/7 days, and birth weight ≤ 1500 g Aged < 1 year, gestational age ≤ 28 weeks, 6/7 days Aged < 2 years with BPD Aged < 2 years with hemodynamically significant congenital heart disease and with: - overt cardiac failure despite pharmacological treatment, or - moderate to severe secondary pulmonary hypertension, or - cyanotic heart disease and arterial oxidation < 90%

Abbreviations: BPD—bronchopulmonary dysplasia.

## Data Availability

The data presented in this study come from the publications summarized in Table 2. The data from the National Institute of Hygiene can be found here: http://wwwold.pzh.gov.pl/oldpage/epimeld/2023/index_mp.html (accessed on 30 April 2023).

## Abbreviations

ARTI	acute respiratory tract infection
COVID-19	coronavirus disease 2019
EMA	European Medicines Agency
HFNC	high-flow nasal cannula
ICD-10	International Classification of Diseases, Tenth Revision
PCR	polymerase chain reaction
RSV	respiratory syncytial virus

## References

[1] De Conto F., Conversano F., Medici M.C., Ferraglia F., Pinardi F., Arcangeletti M.C., Chezzi C., Calderaro A. (2019). Epidemiology of human respiratory viruses in children with acute respiratory tract infection in a 3-year hospital-based survey in Northern Italy. Diagn. Microbiol. Infect. Dis..

[2] Tabatabai J., Ihling C.M., Manuel B., Rehbein R.M., Schnee S.V., Hoos J., Pfeil J., Grulich-Henn J., Schnitzler P. (2023). Viral Etiology and Clinical Characteristics of Acute Respiratory Tract Infections in Hospitalized Children in Southern Germany (2014–2018). Open Forum Infect. Dis..

[3] Takashima M.D., Grimwood K., Sly P.D., Lambert S.B., Chappell K.J., Watterson D., Ware R.S. (2021). Epidemiology of respiratory syncytial virus in a community birth cohort of infants in the first 2 years of life. Eur. J. Pediatr..

[4] Rząd M., Kanecki K., Lewtak K., Tyszko P., Szwejkowska M., Goryński P., Nitsch-Osuch A. (2022). Human respiratory syncytial virus infections among hospitalized children in Poland during 2010–2020: Study based on the national hospital registry. J. Clin. Med..

[5] Glezen W.P., Taber L.H., Frank A.L., Kasel J.A. (1986). Risk of primary infection and reinfection with respiratory syncytial virus. Am. J. Dis. Child..

[6] Nair H., Nokes D.J., Gessner B.D., Dherani M., Madhi S.A., Singleton R.J., O’Brien K.L., Roca A., Wright P.F., Bruce N. (2010). Global burden of acute lower respiratory infections due to respiratory syncytial virus in young children: A systematic review and meta-analysis. Lancet.

[7] Li Y., Wang X., Blau D.M., Caballero M.T., Feikin D.R., Gill C.J., Madhi S.A., Omer S.B., Simões E.A.F., Campbell H. (2022). Global, regional, and national disease burden estimates of acute lower respiratory infections due to respiratory syncytial virus in children younger than 5 years in 2019: A systematic analysis. Lancet.

[8] Black C.P. (2003). Systematic review of the biology and medical management of respiratory syncytial virus infection. Respir. Care.

[9] Crowe J.E., Kliegman R.M., Stanton B.F., St. Geme J.W., Schor N.F. (2016). Respiratory syncytial virus. Nelson Textbook of Pediatrics.

[10] Rha B., Curns A.T., Lively J.Y., Campbell A.P., Englund J.A., Boom J.A., Azimi P.H., Weinberg G.A., Staat M.A., Selvarangan R. (2020). Respiratory syncytial virus-associated hospitalizations among young children: 2015–2016. Pediatrics.

[11] Hall C.B., Weinberg G.A., Iwane M.K., Blumkin A.K., Edwards K.M., Staat M.A., Auinger P., Griffin M.R., Poehling K.A., Erdman D. (2009). The burden of respiratory syncytial virus infection in young children. N. Engl. J. Med..

[12] Hall C.B., Weinberg G.A., Blumkin A.K., Edwards K.M., Staat M.A., Schultz A.F., Poehling K.A., Szilagyi P.G., Griffin M.R., Williams J.V. (2013). Respiratory syncytial virus-associated hospitalizations among children less than 24 months of age. Pediatrics.

[13] Lange J., Kozielski J., Bartolik K., Kabicz P., Targowski T. (2020). Analysis of the incidence of acute respiratory diseases in the paediatric population in Poland in the light of the “Health Needs Map”. Adv. Respir. Med..

[14] Cegielska K., Pogonowska M., Kalicki B. (2018). An analysis of respiratory syncytial virus infections in children up to 24 months old hospitalized in the Department of Paediatrics, Paediatric Nephrology and Allergology of the Military Institute of Medicine between 2016 and 2017. Pediatr. Med. Rodz..

[15] Pancer K., Ciaćka A., Gut W., Lipka B., Mierzejewska J., Milewska-Bobula B., Smorczewska-Kiljan A., Jahnz-Rózyk K., Dzierzanowska D., Madaliński K. (2011). Infections caused by RSV among children and adults during two epidemic seasons. Pol. J. Microbiol..

[16] Pancer K.W., Gut W., Abramczuk E., Lipka B., Litwińska B. (2014). Non-influenza viruses in acute respiratory infections among young children. High prevalence of HMPV during the H1N1V.2009 pandemic in Poland. Przegl. Epidemiol..

[17] Wrotek A., Czajkowska M., Jackowska T. (2020). Seasonality of Respiratory Syncytial Virus Hospitalization. Adv. Exp. Med. Biol..

[18] Homaira N., Oei J.L., Mallitt K.A., Abdel-Latif M.E., Hilder L., Bajuk B., Lui K., Ferson M., Nurkic A., Chambers G.M. (2016). High burden of RSV hospitalization in very young children: A data linkage study. Epidemiol. Infect..

[19] Stein R.T., Bont L.J., Zar H., Polack F.P., Park C., Claxton A., Borok G., Butylkova Y., Wegzyn C. (2017). Respiratory syncytial virus hospitalization and mortality: Systematic review and meta-analysis. Pediatr. Pulmonol..

[20] Del Riccio M., Spreeuwenberg P., Osei-Yeboah R., Johannesen C.K., Vazquez Fernandez L., Teirlinck A.C., Wang X., Heikkinen T., Bangert M., Caini S. (2023). Defining the Burden of Disease of RSV in the European Union: Estimates of RSV-associated hospitalisations in children under 5 years of age. A systematic review and modelling study. J. Infect. Dis..

[21] Wrotek A., Czajkowska M., Jackowska T. (2020). Nosocomial infections in patients hospitalized with respiratory syncytial virus: A practice review. Adv. Exp. Med. Biol..

[22] Pogonowska M., Guzek A., Goscinska A., Rustecka A., Kalicki B. (2022). Compensatory epidemic of RSV infections during the COVID-19 pandemic. An analysis of infections in children hospitalised in the Department of Paediatrics, Paediatric Nephrology and Allergology of the Military Medical Institute in Warsaw in 2020–2021. Pediatr. Med. Rodz.-Paediatr. Fam. Med..

[23] Mapa Potrzeb Zdrowotnych w Zakresie Lecznictwa Szpitalnego dla Polski. https://mpz.mz.gov.pl/wp-content/uploads/2019/06/17_polska.pdf.

[24] Qiu W., Zheng C., Huang S., Zhang Y., Chen Z. (2022). Epidemiological trend of RSV infection before and during COVID-19 pandemic: A three-year consecutive study in China. Infect. Drug Resist..

[25] Casalegno J.S., Ploin D., Cantais A., Masson E., Bard E., Valette M., Fanget R., Targe S.C., Myar-Dury A.F., Doret-Dion M. (2021). Characteristics of the delayed respiratory syncytial virus epidemic, 2020/2021, Rhône Loire, France. Euro Surveill.

[26] Fourgeaud J., Toubiana J., Chappuy H., Delacourt C., Moulin F., Parize P., Scemla A., Abid H., Leruez-Ville M., Frange P. (2021). Impact of public health measures on the post-COVID-19 respiratory syncytial virus epidemics in France. Eur. J. Clin. Microbiol. Infect. Dis..

[27] Pruccoli G., Castagno E., Raffaldi I., Denina M., Barisone E., Baroero L., Timeus F., Rabbone I., Monzani A., Terragni G.M. (2023). The importance of RSV epidemiological surveillance: A multicenter observational study of RSV infection during the COVID-19 pandemic. Viruses.

[28] Cieślak K., Kowalczyk D., Szymański K., Hallmann-Szelińska E., Brydak L.B. (2018). Influenza and influenza-like viruses: Frequent infections in children under 14 years of age during the 2016/2017 epidemic season. Adv. Exp. Med. Biol..

[29] Hallmann-Szelińska E., Bednarska K., Kondratiuk K., Rabczenko D., Brydak L.B. (2016). Viral infections in children in the 2014/2015 epidemic season in Poland. Adv. Exp. Med. Biol..

[30] Woźniak-Kosek A., Czarkowski M.P., Staszewska E., Kondej B., Brydak L.B. (2012). Grypa w Polsce w 2010 roku. Przegl. Epidemiol..

[31] Kondratiuk K., Czarkowski M.P., Hallmann-Szelińska E., Staszewska E., Bednarska K., Cielebąk E., Brydak L.B. (2016). Influenza in Poland in 2013 and 2013/2014 epidemic season. Przegl. Epidemiol..

[32] Bednarska K., Hallmann-Szelińska E., Kondratiuk K., Brydak L.B. (2015). Evaluation of the activity of influenza and influenza-like viruses in the epidemic season 2013/2014. Adv. Exp. Med. Biol..

[33] Bednarska K., Hallmann-Szelińska E., Kondratiuk K., Brydak L.B. (2016). Antigenic drift of A/H3N2/virus and circulation of influenza-like viruses during the 2014/2015 influenza season in Poland. Adv. Exp. Med. Biol..

[34] Byambasuren S., Paradowska-Stankiewicz I., Brydak L.B. (2020). Epidemic influenza seasons from 2008 to 2018 in Poland: A focused review of virological characteristics. Adv. Exp. Med. Biol..

[35] Czarkowski M.P., Hallmann-Szelińska E., Staszewska E., Bednarska K., Kondratiuk K., Brydak L.B. (2014). Influenza in Poland in 2011-2012 and in 2011/2012 and 2012/2013 epidemic seasons. Przegl. Epidemiol..

[36] Łuniewska K., Szymański K., Hallmann-Szelińska E., Kowalczyk D., Sałamatin R., Masny A., Brydak L.B. (2019). Infections caused by influenza viruses among children in Poland during the 2017/18 epidemic season. Adv. Exp. Med. Biol..

[37] Liczba zachorowań i zapadalność na 100 tys. ludności., Zakład Epidemiologii Chorób Zakaźnych i Nadzoru NIZP PZH—PIB Departament Przeciwepidemiczny i Ochrony Sanitarnej Granic GIS, Choroby zakaźne i zatrucia w Polsce—2023 r. http://wwwold.pzh.gov.pl/oldpage/epimeld/2023/index_mp.html.

[38] Xing Y., Proesmans M. (2019). New therapies for acute RSV infections: Where are we?. Eur. J. Pediatr..

[39] Hryniewicz W., Albrecht P., Radzikowski A. (2016). Rekomendacje Postępowania w Pozaszpitalnych Zakażeniach Układu Oddechowego.

[40] Franklin D., Babl F.E., Schlapbach L.J., Oakley E., Craig S., Neutze J., Furyk J., Fraser J.F., Jones M., Whitty J.A. (2018). A randomized trial of high-flow oxygen therapy in infants with bronchiolitis. N. Engl. J. Med..

[41] Franklin D., Shellshear D., Babl F.E., Hendrickson R., Williams A., Gibbons K., McEnery K., Kennedy M., Pham T.M., Acworth J. (2021). High flow in children with respiratory failure: A randomised controlled pilot trial—A paediatric acute respiratory intervention study. J. Paediatr. Child. Health.

[42] Kwon J.W. (2020). High-flow nasal cannula oxygen therapy in children: A clinical review. Clin. Exp. Pediatr..

[43] Ralston S.L., Lieberthal A.S., Meissner H.C., Alverson B.K., Baley J.E., Gadomski A.M., Johnson D.W., Light M.J., Maraqa N.F., Mendonca E.A. (2014). Clinical practice guideline: The diagnosis, management, and prevention of bronchiolitis. Pediatrics.

[44] Fedorczak A., Zielińska N., Nosek-Wasilewska P., Mikołajczyk K., Lisiak J., Zeman K., Tkaczyk M. (2022). Comparison of COVID-19 and RSV infection courses in infants and children under 36 months hospitalized in paediatric department in fall and winter season 2021/2022. J. Clin. Med..

[45] Sosnowska J., Konarska Z., Feleszko W. Real life management of RSV bronchiolitis and comparison with existing practice guidelines. Proceedings of the European Academy of Allergy and Clinical Immunology Congress.

[46] Haładaj K., Fijałkowski B., Chlebna-Sokół D. (2011). Clinical aspects and treatment of respiratory syncytial virus infection in infants in the first 6 months of life. Przegl. Epidemiol..

[47] Wrotek A., Czajkowska M., Jackowska T. (2019). Antibiotic treatment in patients with bronchiolitis. Adv. Exp. Med. Biol..

[48] Wrotek A., Czajkowska M., Jackowska T. (2019). Chest radiography in children hospitalized with bronchiolitis. Adv. Exp. Med. Biol..

[49] Kusak B., Grzesik E., Konarska-Gabryś K., Pacek Z., Piwowarczyk B., Lis G. (2018). Bronchiolitis in children—Do we choose wisely?. Dev. Period Med..

[50] Rogovik A.L., Carleton B., Solimano A., Goldman R.D. (2010). Palivizumab for the prevention of respiratory syncytial virus infection. Can. Fam. Physician.

[51] Helwich E., Miszczak-Knecht M., Sands D., Emich-Widera E. (2022). Stanowisko ekspertów dotyczące rozszerzenia wskazań do profilaktyki ciężkich infekcji wirusem syncytium nabłonka oddechowego (RSV) za pomocą paliwizumabu u noworodków i niemowląt. Stand. Med./Pediatr..

[52] Keam S.J. (2023). Nirsevimab: First Approval. Drugs.

[53] Hammitt L.L., Dagan R., Yuan Y., Baca Cots M., Bosheva M., Madhi S.A., Muller W.J., Zar H.J., Brooks D., Grenham A. (2022). Nirsevimab for prevention of RSV in healthy late-preterm and term infants. N. Engl. J. Med..

[54] Álvarez García F.J., Cilleruelo Ortega M.J., Álvarez Aldeán J., Garcés-Sánchez M., Garrote Llanos E., Iofrío de Arce A., Montesdeoca Melián A., Navarro Gómez M.L., Pineda Solas V., Rivero Calle I. (2023). Immunisation schedule of the Spanish Association of Paediatrics: 2023 Recommendations. An. Pediatr. (Engl. Ed.).

[55] Stratégie de Prévention des Bronchiolites à VRS des Nourrissons Avis des Sociétés Savantes Françaises de Pédiatrie. https://www.infovac.fr/docman-marc/public/bulletins/2023/1869-lien-1-strategie-de-prevention-des-bronchiolites-a-vrs-des-nourrissons/file.

[56] Sun M., Lai H., Na F., Li S., Qiu X., Tian J., Zhang Z., Ge L. (2023). Monoclonal Antibody for the Prevention of Respiratory Syncytial Virus in Infants and Children A Systematic Review and Network Meta-analysis. JAMA Netw. Open.

[57] Chuang Y.C., Lin K.P., Wang L.A., Yeh T.K., Liu P.Y. (2023). The impact of the COVID-19 pandemic on respiratory syncytial virus infection: A narrative review. Infect. Drug Resist..

[58] Eden J.S., Sikazwe C., Xie R., Deng Y.M., Sullivan S.G., Michie A., Levy A., Cutmore E., Blyth C.C., Britton P.N. (2022). Off-season RSV epidemics in Australia after easing of COVID-19 restrictions. Nat. Commun..

[59] Garg I., Shekhar R., Sheikh A.B., Pal S. (2022). Impact of COVID-19 on the changing patterns of respiratory syncytial virus infections. Infect. Dis. Rep..

[60] Wang L., Davis P.B., Berger N.A., Kaelber D.C., Volkow N.D., Xu R. (2022). Disruption in seasonality, patient characteristics and disparities of respiratory syncytial virus infection among young children in the US during and before the COVID-19 pandemic: 2010–2022. medRxiv.

[61] Bermúdez Barrezueta L., Matías Del Pozo V., López-Casillas P., Brezmes Raposo M., Gutiérrez Zamorano M., Pino Vázquez M.A. (2022). Variation in the seasonality of the respiratory syncytial virus during the COVID-19 pandemic. Infection.

[62] Bozzola E., Barni S., Villani A. (2022). Respiratory syncytial virus pediatric hospitalization in the COVID-19 era. Int. J. Environ. Res. Public Health.

[63] Istel M. (2023). Zakażenia RSV: Jak ich przybywa w Polsce i Europie. Konkret 24. https://konkret24.tvn24.pl/zdrowie/grypa-rsv-koronawirus-zakazenia-rsv-jak-przybywa-w-polsce-i-europie-6592401.

[64] Wrotek A., Badyda A., Czechowski P.O., Owczarek T., Dąbrowiecki P., Jackowska T. (2021). Air pollutants’ concentrations are associated with increased number of RSV hospitalizations in Polish children. J. Clin. Med..

[65] Jenkins V.A., Hoet B., Hochrein H., De Moerlooze L. (2023). The quest for a respiratory syncytial virus vaccine for older adults: Thinking beyond the F protein. Vaccines.

[66] Obando-Pacheco P., Justicia-Grande A.J., Rivero-Calle I., Rodríguez-Tenreiro C., Sly P., Ramilo O., Mejías A., Baraldi E., Papadopoulos N.G., Nair H. (2018). Respiratory syncytial virus seasonality: A global overview. J. Infect. Dis..

[67] Li Y., Wang X., Broberg E.K., Campbell H., Nair H., European RSV Surveillance Network (2022). Seasonality of respiratory syncytial virus and its association with meteorological factors in 13 European countries, week 40 2010 to week 39 2019. Euro Surveill.

